# Unfavorable Contribution of a Tissue-Engineering Cartilage Graft to Osteochondral Defect Repair in Young Rabbits

**DOI:** 10.3389/fcell.2020.595518

**Published:** 2020-10-29

**Authors:** Zhihua Lu, Sheng Zhou, Justin Vaida, Gongming Gao, Amanda Stewart, Joshua Parenti, Lianqi Yan, Ming Pei

**Affiliations:** ^1^Stem Cell and Tissue Engineering Laboratory, Department of Orthopaedics, West Virginia University, Morgantown, WV, United States; ^2^Department of Orthopedics, Clinical Medical College of Yangzhou University, Subei People’s Hospital of Jiangsu Province, Yangzhou, China; ^3^WVU Cancer Institute, Robert C. Byrd Health Sciences Center, West Virginia University, Morgantown, WV, United States

**Keywords:** young rabbit, osteochondral defect, tissue engineering, decellularized extracellular matrix, infrapatellar fat pad-derived stem cell

## Abstract

A stem cell-based tissue-engineering approach is a promising strategy for treatment of cartilage defects. However, there are conflicting data in the feasibility of using this approach in young recipients. A young rabbit model with an average age of 7.7 months old was used to evaluate the effect of a tissue-engineering approach on the treatment of osteochondral defects. Following *in vitro* evaluation of proliferation and chondrogenic capacity of infrapatellar fat pad-derived stem cells (IPFSCs) after expansion on either tissue culture plastic (TCP) or decellularized extracellular matrix (dECM), a premature tissue construct engineered from pretreated IPFSCs was used to repair osteochondral defects in young rabbits. We found that dECM expanded IPFSCs exhibited higher proliferation and chondrogenic differentiation compared to TCP expanded cells in both pellet and tissue construct culture systems. Six weeks after creation of bilateral osteochondral defects in the femoral trochlear groove of rabbits, the Empty group (left untreated) had the best cartilage resurfacing with the highest score in Modified O’Driscoll Scale (MODS) than the other groups; however, this score had no significant difference compared to that of 15-week samples, indicating that young rabbits stop growing cartilage once they reach 9 months old. Interestingly, implantation of premature tissue constructs from both dECM and TCP groups exhibited significantly improved cartilage repair at 15 weeks compared to those at six weeks (about 9 months old), indicating that a tissue-engineering approach is able to repair adult cartilage defects. We also found that implanted pre-labeled cells in premature tissue constructs were undetectable in resurfaced cartilage at both time points. This study suggests that young rabbits (less than 9 months old) might respond differently to the classical tissue-engineering approach that is considered as a potential treatment for cartilage defects in adult rabbits.

## Introduction

Articular cartilage holds a limited capacity for self-healing due to a shortage of blood supply. Several surgical methods are available for the treatment of cartilage damage, including arthroscopic debridement, microfracture, and osteochondral transplantation; (Willers et al., 2003) however, none can consistently reproduce normal hyaline cartilage (Smith et al., 2005). As an alternative treatment, stem cell-based tissue engineering has been validated as a promising approach to reconstitute cartilage defects (Nukavarapu and Dorcemus, 2013). Seed cells and scaffolds are two important parameters for the success of a tissue-engineering strategy. Increasing data indicate the advantages of infrapatellar fat pad (IPFP)-derived stem cells (IPFSCs) as a stem cell source due to strong proliferation capacities and multilineage differentiation potentials, particularly for cartilage engineering and regeneration (Sun et al., 2018; Wang T. et al., 2020). Among the candidate scaffold materials, polylactic-co-glycolic acid (PLGA) is one of the most widely used biodegradable polymers, owing to its prominent advantages such as maneuverability of degradation rates and outstanding processability (Uematsu et al., 2005). Therefore, in this study, IPFSCs were chosen as seed cells to grow on PLGA scaffolds.

Cell expansion on a two-dimensional (2D) culture substrate often causes stem cell senescence (Li and Pei, 2012). Evidence indicates that decellularized extracellular matrix (dECM), a three-dimensional (3D) culture system, can efficiently rejuvenate expanded stem cells in both proliferation and chondrogenic differentiation (Li and Pei, 2010; Pei et al., 2011; Pei, 2017). A previous report successfully utilized dECM expanded synovium-derived stem cells in the treatment of partial-thickness cartilage defects in a minipig model *via* intraarticular injection (Pei et al., 2013). Given that a stem cell-based tissue-engineering approach exhibits a promising strategy to overcome the challenge of tissue defects in elderly recipients, (Uematsu et al., 2005; Han et al., 2008) there are few reports available to determine the feasibility of this approach in cartilage repair in young recipients, considering that older transplant recipients exhibited differently from young recipients in some biological aspects such as in immunosenescence (Colvin et al., 2017). Moreover, there is no consensus on skeletally mature rabbit age with a range from four to nine months old (Masoud et al., 1986; Wei et al., 1997; Wei and Messner, 1999; Rudert, 2002; Reinholz et al., 2004; Hoemann et al., 2007; Hunziker et al., 2007; Pei et al., 2009; Isaksson et al., 2010). In this study, a rabbit model (between 7.5–8 months old) considered as skeletally mature (Masoud et al., 1986; Gilsanz et al., 1988; Newman et al., 1995) was used to evaluate whether articular cartilage became mature and whether a tissue-engineering approach benefited the treatment of osteochondral defects. We hypothesized that a young rabbit (less than 9 months old) does not have mature cartilage and may not respond to a tissue-engineering approach for cartilage repair the same as an adult rabbit does.

## Materials and Methods

### Experimental Design

**FIGURE 1:**
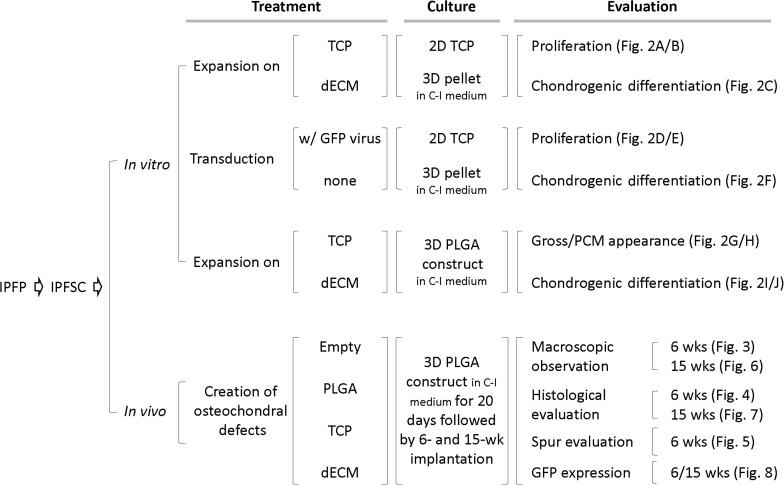
Schematic diagram of experimental design. Abbreviation: C-I – chondrogenic induction; dECM – decellularized extracellular matrix; GFP – green fluorescence protein; PCM – phase contrast microscopy; TCP – tissue culture plastic.

**FIGURE 2:**
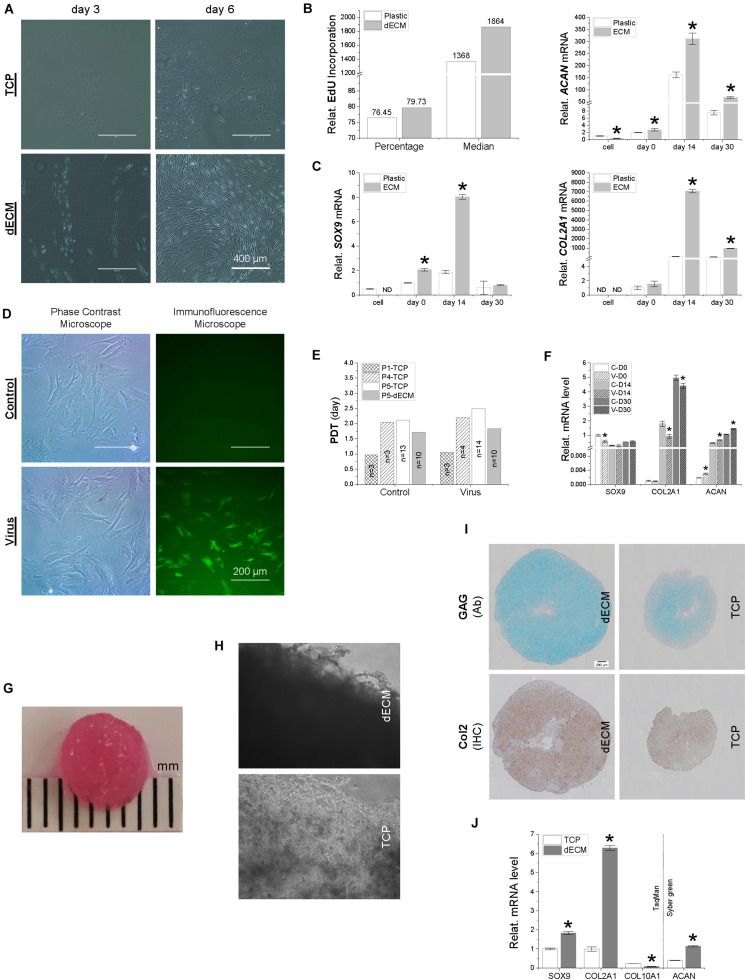
A comparison of IPFSCs and their capacity for proliferation and chondrogenic differentiation following expansion on either dECM or TCP **(A–C)** and with or without lentivirus transduction **(D**–**F)** as well as of premature tissue constructs seeded with 2.2 × 10^6^ IPFSCs after expansion on dECM or TCP **(G**–**K)**. **(A)** Cell morphology of IPFSCs after 3- and 6-day culture on either dECM or TCP; Scale bar: 400 μm; **(B)** relative EdU incorporation of dECM or TCP expanded IPFSCs (5 × 10^5^) measured by flow cytometry, and **(C)** RT-qPCR evaluation of expression level of chondrogenic marker genes (*SOX9*, *ACAN*, and *COL2A1*) in dECM or TCP expanded IPFSCs (*n* = 4) after 30-day chondrogenic induction in a pellet culture system. *p < 0.05 as compared to the control group (TCP). **(D)** Transduction efficiency in puromycin screened IPFSCs visualized by immunofluorescence and phase contrast microscopy; Scale bar: 200 μm; **(E)** population doubling time (PDT) in IPFSCs with or without transduction following dECM and TCP expansion; and **(F)** expression of chondrogenic marker genes (*SOX9*, *ACAN*, and *COL2A1*) *via* RT-qPCR in IPFSCs (*n* = 4) with (“V”) or without (“C”) transduction after 30-day chondrogenic induction in a pellet culture system. *p < 0.05 as compared to the control group (non-virus transduction). **(G)** A representative photo of a two-week tissue construct; **(H)** phase contrast microscopy of 20-day tissue constructs (dECM or TCP expanded IPFSCs grown on PLGA mesh); **(I)** histological evaluation of 20-day tissue constructs using Alcian blue staining (Ab) for sulfated GAGs and immunohistochemical staining (IHC) for type II collagen; Scale bar: 200 μm; **(J)** expression of chondrogenic marker genes (*SOX9*, *ACAN*, *COL2A1*, and *COL10A1*) *via* RT-qPCR analysis in dECM or TCP expanded IPFSCs (*n* = 4) after 20-day chondrogenic induction in six-well plates. *p < 0.05 as compared to the control group (TCP).

**FIGURE 3:**
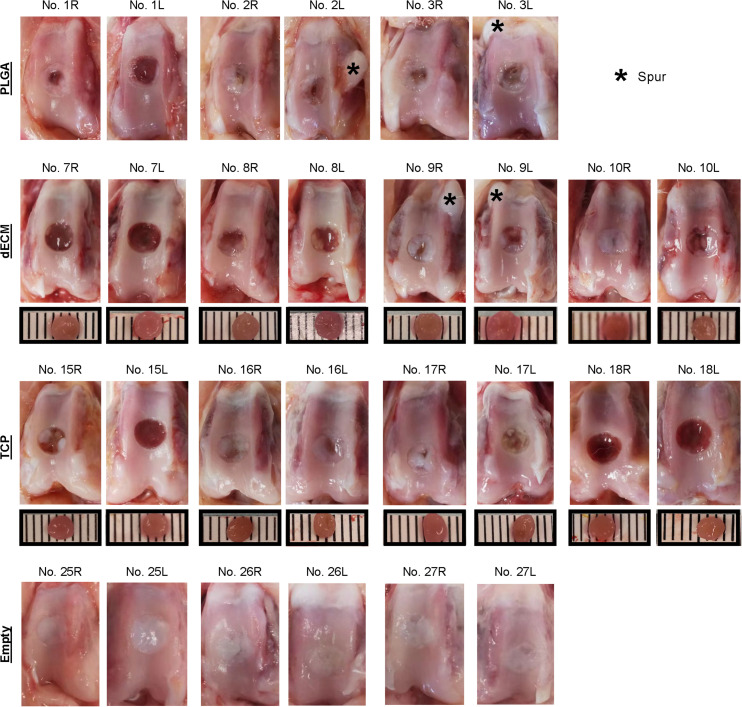
Macroscopic observation of six-week osteochondral defects repaired with PLGA mesh alone (PLGA; *n* = 3 rabbits/6 knees), tissue constructs developed from dECM expanded IPFSCs (dECM; *n* = 4 rabbits/8 knees) or TCP expanded cells (TCP; *n* = 4 rabbits/8 knees), or left untreated (Empty; *n* = 3 rabbits/6 knees). Scale bar: 1 mm.

**FIGURE 4:**
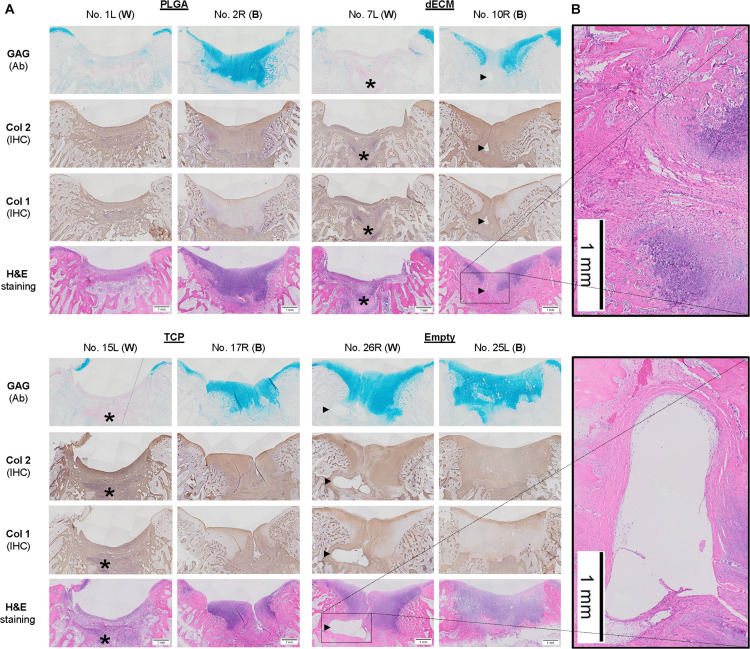
Histological evaluation of six-week osteochondral defects repaired with PLGA mesh alone (PLGA; *n* = 6 knees), tissue constructs developed from dECM expanded IPFSCs (dECM; *n* = 8 knees) or TCP expanded cells (TCP; *n* = 8 knees), or left untreated (Empty; *n* = 6 knees) using Alcian blue staining (Ab) for sulfated GAGs, H&E staining for the intact tidemark, and immunohistochemical staining (IHC) for types I and II collagen (Col 1 and Col 2). **(A)** Two representative cartilage resurfacings were chosen from each group to serve as the best repair (“B”) including rabbit No. 2R/10R/17R/25L or the worst repair (“W”) including rabbit No. 1L/7L/15L/26R. Arrows (▶) indicate location of subchondral bone cysts and the asterisk (*) indicates mononuclear cells. **(B)** Bone cysts were shown at higher magnification in H&E staining. Scale bar: 1 mm.

**FIGURE 5:**
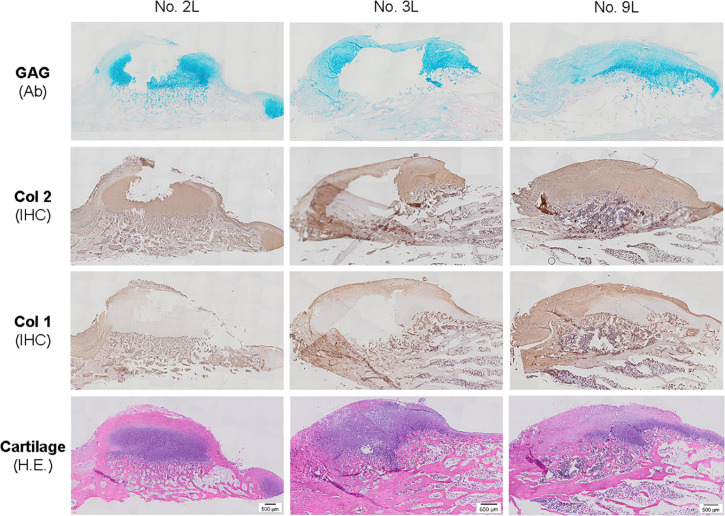
Histological evaluation of bone spurs in 6-week cartilage resurfacing using Alcian blue staining (Ab) for sulfated GAGs, H&E staining for cartilage tissue and immunohistochemical staining (IHC) for types I and II collagen (Col 1 and Col 2). Scale bar: 500 μm.

**FIGURE 6:**
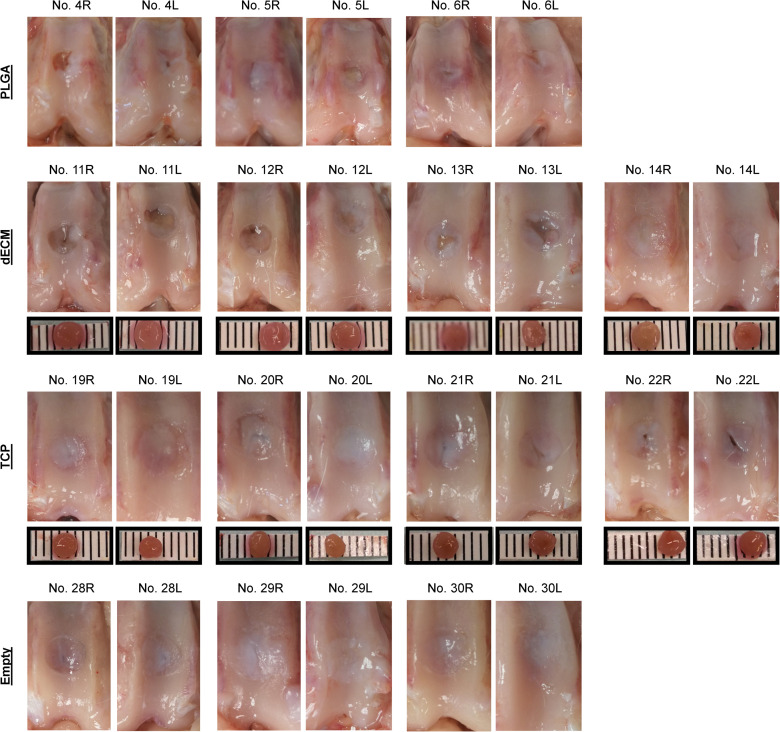
Macroscopic observation of 15-week osteochondral defects repaired with PLGA mesh alone (PLGA; *n* = 3 rabbits/6 knees), tissue constructs developed from dECM expanded IPFSCs (dECM; *n* = 4 rabbits/8 knees) or TCP expanded cells (TCP; *n* = 4 rabbits/8 knees), or left untreated (Empty; *n* = 3 rabbits/6 knees). Scale bar: 1 mm.

**FIGURE 7:**
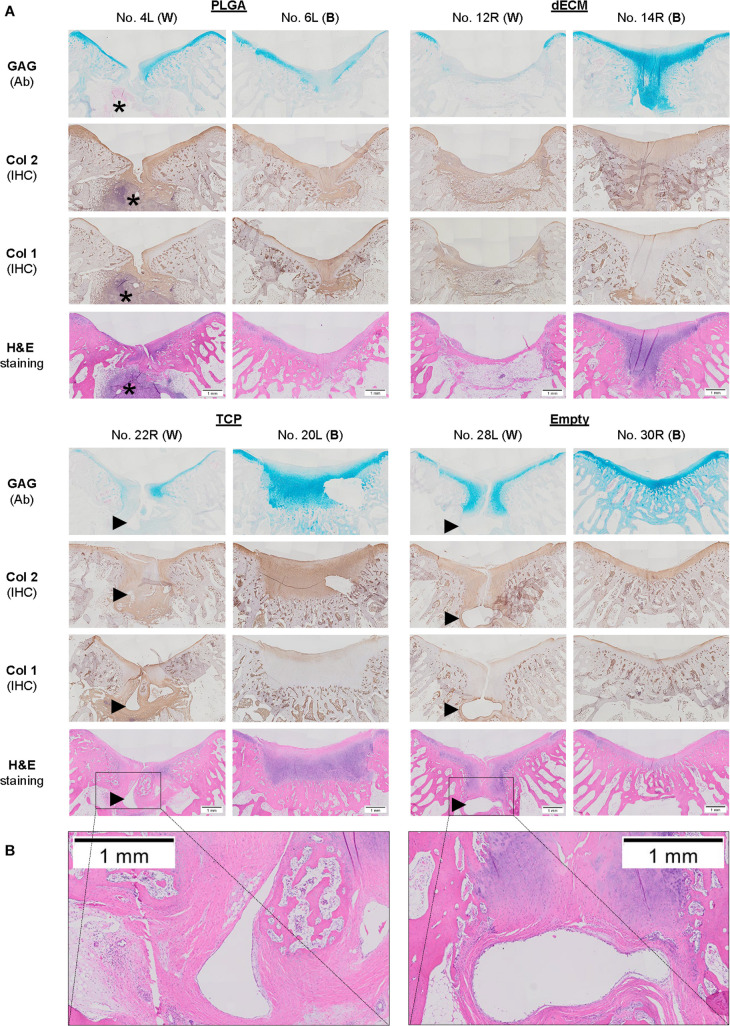
Histological evaluation of 15-week osteochondral defects repaired with PLGA mesh alone (PLGA; *n* = 6 knees), tissue constructs developed from dECM expanded IPFSCs (dECM; *n* = 8 knees) or TCP expanded cells (TCP; *n* = 8 knees), or left untreated (Empty; *n* = 6 knees) using Alcian blue staining (Ab) for sulfated GAGs, H&E staining for the intact tidemark, and immunohistochemical staining (IHC) for types I and II collagen (Col 1 and Col 2). **(A)** Two representative cartilage resurfacings were chosen from each group to serve as the best repair (“B”) including rabbit No. 6L/14R/20L/30R or the worst repair (“W”) including rabbit No. 4L/12R/22R/28L. Arrows (▶ indicate location of subchondral bone cysts and the asterisk (*) indicates inflammatory cells. **(B)** Bone cysts were shown at higher magnification in H&E staining. Scale bar: 1 mm.

**FIGURE 8:**
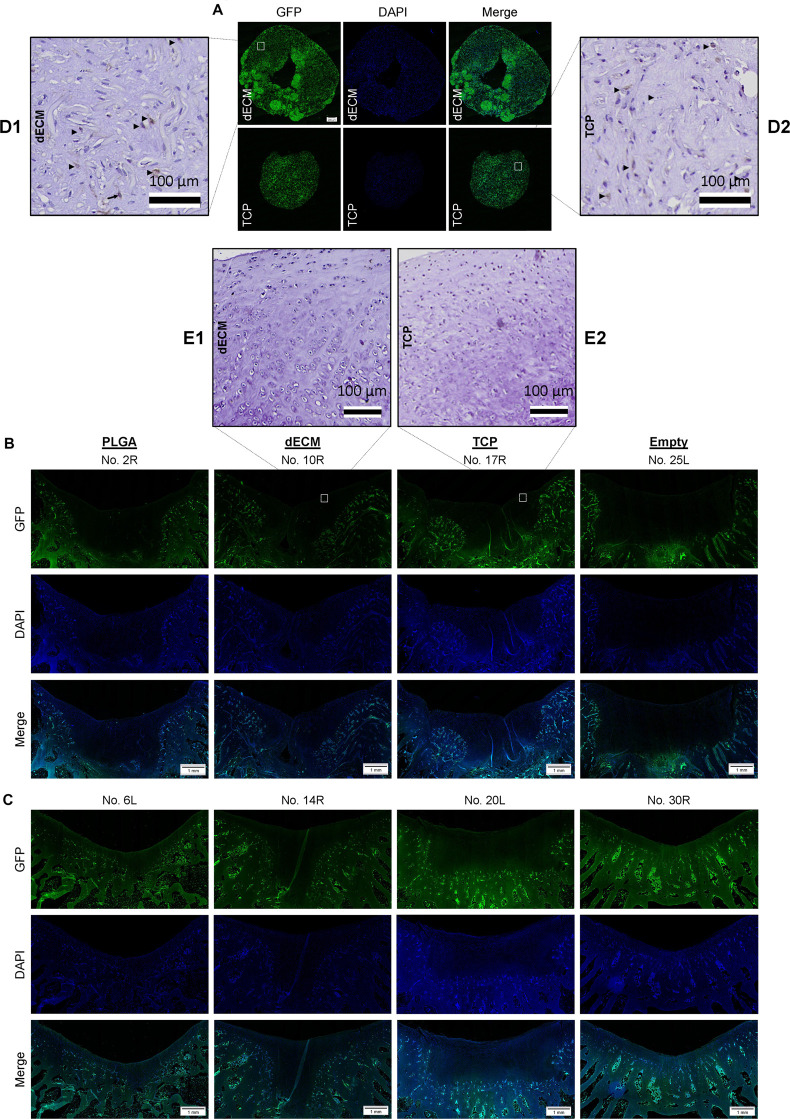
Track of implanted cells labeled with GFP signal. Immunofluorescence of GFP expression in *in vitro* tissue constructs (Scale bar: 200 μm) from either dECM or TCP expanded IPFSCs **(A)**, and six-week (**(B)** and 15-week **(C)** osteochondral defects repaired with PLGA mesh alone (PLGA; *n* = 6 knees), tissue constructs developed from dECM expanded IPFSCs (dECM; *n* = 8 knees) or TCP expanded cells (TCP; *n* = 8 knees), or left untreated (Empty; *n* = 6 knees) (Scale bar: 1 mm). DAPI served as a counterstain. Immunohistochemical staining using monoclonal antibody showed positive staining (Arrows; ▶) for *in vitro* tissue constructs **(D)** but negative staining for *in vivo* resurfacing cartilage from the tissue construct groups **(E)**. Scale bar: 100 μm. Hematoxylin served as a counterstain.

Following isolation of IPFSCs from rabbit IPFP, both *in vitro* and *in vivo* studies were designed (Figure 1). In the *in vitro* study (Figure 2), IPFSCs were evaluated in cell proliferation and chondrogenic differentiation (3D culture systems - both pellets and PLGA tissue constructs) by comparing the influence of (1) dECM expansion with tissue culture plastic (TCP) as a control and (2) lentivirus transduction with non-transduction as a control. In the *in vivo* study (Figures 3–8), after creation of osteochondral defects, four groups were designed: Empty group (left untreated), PLGA group (filled with PLGA alone), TCP group (filled with 20-day-cartilage grafts using TCP expanded IPFSCs), and dECM group (filled with 20-day-cartilage grafts using dECM expanded IPFSCs). Histological evaluation was quantified for cartilage resurfacing of osteochondral defects (Tables 1–3) and implanted cells were tracked using both immunofluorescence microscopy and immunohistochemical staining for green fluorescence protein (GFP) (Figure 8).

**TABLE 1:** Modified O’Driscoll histological scoring system.

Category	Score
**I: Percentage of repair tissue that is hyaline cartilage**	
100–125%	6
80–100%	8
60–80%	6
40–60%	4
20–40%	2
0–20%	0
**II: Articular surface continuity**	
Continuous and smooth	2
Continuous but rough	1
Discontinuous	0
**III: Tidemark**	
Present	2
Incomplete (degenerative, vessel crossing)	1
Absent	0
**IV: Thickness of repair tissue compared to host cartilage**	
121–150% of normal cartilage	1
81–120% of normal cartilage	2
51–80% of normal cartilage	1
0–50% of normal cartilage	0
**V: Integration of cartilage**	
Complete (integrated at both sides)	2
Partial	1
Poor (not integrated at both sides)	0
**VI: Degenerated changes in repair tissue**	
Normal cellularity	2
Slight to moderate hypocellularity or hypercellularity	1
Severe hypocellularity or hypercellularity	0
**VII: Degenerative changes in adjacent cartilage**	
Normal cellularity, no clusters, no fibrillations	3
Normal cellularity, mild clusters, superficial fibrillations	2
Mild or moderate changes in cellularity, moderate fibrillations	1
Severe changes in cellularity, severe fibrillations	0
**VIII: Chondrocyte clustering**	
No clusters	2
<25% of the cells	1
25–100% of the cells	0
**Total**	**Max. 23**

**TABLE 2:** Adaptive reactions in cartilage resurfacing.

Group	Category			
	*Bone cyst*		*Mononuclear cells*	
6 weeks	No.	Ratio	No.	Ratio
PLGA	3L	1/6	–	–
dECM	10R	1/8	7L/7R/8L/8R	4/8
TCP	17R	1/8	15L/15R/18L/18R	4/8
EMPTY	26R	1/6	–	–
**15 weeks**				
PLGA	4L/5R	2/6	4L	1/6
dECM	28L/28R	2/8	12L	1/8
TCP	20R/21L/21R/22L/22R	5/8	–	–
EMPTY	28L/28R	2/6	–	–

**TABLE 3:** Six-week and 15-week cartilage resurfacing graded by MODS.

Group	Category								Total score
	I	II	III	IV	V	VI	VII	VIII	
**6 weeks**									
PLGA	3.33	0.50	0.17	1.17	1.67	0.50	2.33	1.00	10.67 ± 5.82
dECM	2.00	0.50	-	-	1.50	0.63	2.25	0.88	7.75 ± 3.49
TCP	2.25	0.50	0.13	0.50	1.63	0.75	2.00	1.00	8.75 ± 4.83
EMPTY	7.67	2.00	1.00	1.00	1.83	2.00	2.00	1.00	18.50 ± 0.84
**15 weeks**									
PLGA	5.33	1.00	0.83	1.83	1.50	1.83	1.67	1.00	15.00 ± 3.35
dECM	3.75	1.38	0.50	1.13	2.00	1.25	2.00	1.00	13.00 ± 5.61
TCP	5.50	1.25	0.63	1.13	1.75	1.13	2.00	1.00	14.38 ± 3.81
EMPTY	7.67	1.83	1.17	1.67	1.83	1.33	2.00	1.00	18.50 ± 1.38

### IPFSC Isolation and Culture

This animal study was approved by the Institutional Animal Care and Use Committee. Infrapatellar fat pads from four New Zealand White (NZW) rabbits were used to collect stem cells (IPFSCs) after a sequential digestion using 0.1% trypsin (Roche, Indianapolis, IN) for 30 min and 0.1% collagenase P (Roche) for 2 h to release cells. The stemness of IPFSCs was characterized in both human (He and Pei, 2013; Pizzute et al., 2016; Wang et al., 2019; Wang Y. M. et al., 2020) and rabbit donors (Wang T. et al., 2020). The pooled IPFSCs were cultured in growth medium [Minimum Essential Medium–Alpha Modification (αMEM) containing 10% fetal bovine serum (FBS), 100 U/mL penicillin, 100 μg/mL streptomycin, and 0.25 μg/mL fungizone (Invitrogen, Carlsbad, CA)] at 37°C in a humidified 21% O_2_ and 5% CO_2_ incubator. The medium was changed every three days.

### IPFSC Labeling

Passage 2 rabbit IPFSCs were transduced with lentivirus carrying GFP in the presence of 4 μg/mL of protamine sulfate (MilliporeSigma, Burlington, MA). Twenty-four hours later, the medium was replaced with αMEM with 10% FBS and 2 μg/mL of puromycin (MilliporeSigma) for cell screening. Passage 5 rabbit IPFSCs labeled with GFP were collected for the *in vivo* study.

### dECM Preparation

dECM was prepared by following a protocol described in a previous report (Li and Pei, 2018). Briefly, TCP was treated with 0.2% gelatin (MilliporeSigma), 1% glutaraldehyde (MilliporeSigma), and 1 M ethanolamine (MilliporeSigma). Passage 2 IPFSCs at 100% confluence on pre-coated TCP were treated with 250 μM of L-ascorbic acid phosphate (Wako Chemicals, Richmond, VA) for seven days (Pizzute et al., 2016) followed by an incubation with extraction buffer (0.5% Triton X-100 containing 20 mM ammonium hydroxide). After cells were removed, dECM was stored in phosphate buffered solution (PBS) containing 100 U/mL penicillin, 100 μg/mL streptomycin, and 0.25 μg/mL fungizone at 4°C until use.

### Three Experiments Were Designed as Follows

1) **A comparison of dECM and TCP expanded IPFSCs in proliferation and chondrogenic differentiation:** Passage 5 IPFSCs were expanded on TCP and dECM for one passage followed by a 30-day chondrogenic induction in a pellet culture system. Cell morphology and relative 5-Ethynyl-2′-deoxyuridine (EdU) incorporation were evaluated for proliferation capacity. A serum-free chondrogenic medium consisted of high-glucose Dulbecco’s modified Eagle’s medium, 100 nM dexamethasone, 40 μg/mL proline, 0.1 mM L-ascorbic acid-2-phosphate, 100 U/mL penicillin, 100 μg/mL streptomycin, 0.25 μg/mL fungizone, and 1 × ITS^TM^ Premix (Corning, Bedford, MA) with the addition of 10 ng/mL transforming growth factor beta3 (TGF-β3; PeproTech, Rocky Hill, NJ). Real-time quantitative PCR (RT-qPCR) analysis was used to assess mRNA levels of chondrogenic markers [*SOX9* (SRY-box 9), *ACAN* (aggrecan), and *COL2A1* (type II collagen alpha I chain)] in expanded cells and chondrogenic pellets (day 0, 14, and 30).

Following our previously published methods, (Pei et al., 2002a, b) 1.3 × 10^6^ cells from either TCP or dECM expansion were seeded on one 5 mm diameter × 2 mm thickness PLGA mesh (Synthecon, Houston, TX) in a spinner flask. After incubation for 72 h to allow cell attachment, the cell-scaffold constructs were transferred into six-well plates and cultured in a serum-free chondrogenic medium in a standard incubator (5% CO_2_ and 21% O_2_) for ten days and subsequently in a hypoxia incubator (5% CO_2_ and 5% O_2_) for ten days (Li et al., 2011; Galeano-Garces et al., 2017). Constructs were harvested at day 20 for chondrogenic evaluation [*SOX9*, *ACAN*, *COL2A1*, and *COL10A1* (type X collagen alpha1)] using RT-qPCR analyses.

Cell proliferation was evaluated using the Click-iT^TM^ EdU Alexa Fluor^TM^ 647 Flow Cytometry Assay Kit (Invitrogen). IPFSCs (5 × 10^5^) were incubated with 10 μM EdU for 18 h followed by staining as per manufacturer’s protocol. Briefly, cells were incubated with Click-iT^TM^ fixative for 15 min in the dark followed by washing with 1% bovine serum albumin (BSA)-PBS and then resuspended in 1 × Click-iT^TM^ saponin-based permeabilization buffer. Following staining in labeling cocktail for 30 min, cells were analyzed with a FACS Calibur (BD Biosciences, San Jose, CA) and data analyzed using FCS Express software package (*De Novo* Software, Pasadena, CA).

For RT-qPCR, total RNA was extracted from tissue constructs (n = 4) using TRIzol^®^ (Life Technologies, Carlsbad, CA) as per manufacturer’s protocol. Subsequently, cDNA was synthesized from mRNA by reverse transcriptase using a High-Capacity cDNA Archive Kit (Thermo Fisher Scientific, Waltham, MA). Primers of the chondrogenic marker gene [*ACAN* (forward GCTACGGAGACAAGGATGAGTTC and reverse CGTAAAAGACCTCACCCTCCAT)] and endogenous control gene *GAPDH* (glyceraldehyde-3-phosphate dehydrogenase; forward TTCCACGGCACGGTCAAGGC and reverse GGGCAC CAGCATCACCCCAC) were designed by Integrated DNA Technologies (IDT, Coralville, IA) as a SYBR^®^ green gene expression assay using their PCR primer design tool. Primers for chondrogenic-related genes [*SOX9* (Assay ID Oc04096872_m1), *COL2A1* (Assay ID Oc03396132_g1), and *COL10A1* (Assay ID Oc04097225_s1)] were used in a TaqMan^®^ gene expression assay from Applied Biosystems (Foster City, CA). RT-qPCR was performed using the iCycler iQ^TM^ Multicolor RT-PCR Detection.

2) **A comparison of IPFSCs with or without lentivirus transduction in proliferation and chondrogenic differentiation:** Passage 5 IPFSCs with or without transduction of lentivirus vector carrying GFP (pRSC-SFFV-Luciferase-E2A-Puro-E2A-GFP-wpre) were evaluated for potential influence of viral transduction on cell proliferation and chondrogenic capacity. Immunofluorescence microscopy was used to demonstrate successful transduction following puromycin screening. TCP expanded IPFSCs with or without transduction were counted in T175 TCP (n = 3∼14) using a hemocytometer from passage 1 to 5 along with dECM expanded cells at passage 5 with or without transduction. Cell population doubling time (PDT) was then calculated as “PDT = T^∗^log (2)/[log (N_1_) – log (N_0_)]”, where T represents incubation time, N_1_ for harvesting cell number, and N_0_ for plating cell number. Expanded IPFSCs (4 × 10^5^ cells) with or without transduction at passage 5 were pelleted by centrifugation in a 15-ml polypropylene tube at 1200 revolutions per minute for 7 min. Following overnight incubation (day 0 samples), pellets were grown in a serum-free chondrogenic medium for up to 30 days. Pellets were harvested at day 0, 14, and 30 for evaluation of chondrogenic marker genes (*SOX9*, *COL2A1*, and *ACAN*) *via* RT-qPCR.
3) **Using GFP-labeled IPFSCs with or without dECM expansion to develop premature tissue constructs to repair osteochondral defects in young rabbits**. GFP-labeled passage 6 IPFSCs (2.2 × 10^6^ cells) with or without dECM expansion were seeded in 5 mm diameter × 2 mm thickness PLGA mesh in a spinner flask for three days, (Pei et al., 2002a, b) followed by culture in six-well plates in the presence of serum-free chondrogenic induction medium in a standard incubator (5% CO_2_ and 21% O_2_) for ten days and subsequently in a hypoxia incubator (5% CO_2_ and 5% O_2_) for ten days (Li et al., 2011; Galeano-Garces et al., 2017). After observation with immunofluorescence microscopy to confirm the presence of a GFP signal, 20-day tissue constructs developed from either dECM or TCP expanded IPFSCs were used to repair osteochondral defects in young rabbits.

Young NZW rabbits (n = 28, female, 2.5–4 kg, 235.2 ± 2.7 days with an average age of 7.7-months) (Envigo Global Services Inc., Denver, PA) were used in this study. Anesthesia was induced with an intramuscular injection with 5 mg/kg xylazine (Phoenix Pharmaceutical, St. Joseph, MO) and 35 mg/kg ketamine (Phoenix Pharmaceutical) and maintained with isofluorane. The patella was dislocated laterally and a 4.76 mm diameter × 2 mm depth osteochondral defect was created in the patellar groove of the femur in both knees using a custom designed hand drill with a depth stop. Four groups were designated: defects treated with premature tissue constructs developed by either dECM or TCP expanded cells (the dECM group and the TCP group, respectively) (n = 16 knees/8 rabbits/group), and PLGA scaffold only (the PLGA group) or left untreated (the Empty group) (*n* = 12 knee/6 rabbits/group). Six weeks and 15 weeks postoperatively, rabbits in each group were euthanized for gross observation and histologic evaluation for cartilage resurfacing.

For macroscopic evaluation, once both knee joints were opened, the defect area of the patellar groove was photographed, and gross examination was performed. Femoral condyles were dissected followed by fixation in 4% paraformaldehyde in PBS at 4°C for three days. Each specimen was decalcified by incubation in 15% ethylenediaminetetraacetic acid (EDTA)/0.1% paraformaldehyde solution for six weeks. A 5-μm thick section of the grafted area in the coronal plane was stained using Alcian blue (counterstained with fast red) for sulfated glycosaminoglycans (GAGs) and hematoxylin-eosin staining (H&E) for identification of the intact tidemark line that separates calcified and non-calcified cartilaginous matrix. For immunohistochemical analysis, 1% hydrogen peroxide (H_2_O_2_) in methanol was used to inactivate endogenous peroxidase activity. Sections were digested with 2 mg/mL hyaluronidase for 30 min followed by overnight incubation at 4°C with monoclonal mouse antibodies against type I collagen (MilliporeSigma) and type II collagen (Developmental Studies Hybridoma Bank, Iowa City, IA). Sections for GFP detection were treated with citrate unmasking solution for 20 min followed by overnight incubation at 4°C with a monoclonal mouse antibody against GFP (4B10, Cell Signaling Technology, Danvers, MA). After extensive washing with PBS, sections were incubated with a secondary antibody for 30 min at room temperature. Immunostaining conducted with Vectastain^®^ ABC reagent (Vector Laboratories, Burlingame, CA) was followed by 3,30-diaminobenzidine (DAB) staining and counterstaining was performed with hematoxylin (Vector Laboratories). Tissue sections were graded by four experts blinded to group assignment using a Modified O’Driscoll Scale (MODS) (Table 1; O’Driscoll et al., 1986; Rutgers et al., 2010; Barron et al., 2015).

### Statistical Analysis

Results from RT-qPCR and histological scoring are presented as mean ± standard error of the mean; the *t*-test was used to assess data between two groups. All statistical analyses were performed with SPSS 13.0 statistical software (SPSS, Inc., Chicago, IL); *p* < 0.05 was considered statistically significant.

## Results

### dECM Expanded IPFSCs Exhibited Superior Capacity in Proliferation and Chondrogenic Differentiation

To determine whether dECM expansion could rejuvenate IPFSCs’ proliferation and chondrogenic differentiation, IPFSCs were grown on dECM and TCP for one passage followed by chondrogenic induction in a pellet culture system. We found that IPFSCs grown on dECM exhibited a glistening profile and were arranged in the direction of matrix fibers below; in contrast, IPFSCs grown on TCP were larger in size and arranged in a disorderly fashion (Figure 2A). EdU incorporation data showed that dECM expanded IPFSCs had a 4.3% increase in percentage and 36.3% increase in median compared to TCP expanded cells (Figure 2B). After chondrogenic induction, we found that dECM expanded IPFSCs exhibited significantly higher expression levels of chondrogenic marker genes (Figure 2C), including *SOX9*, *ACAN*, and *COL2A1*, than the corresponding TCP group in a time-dependent manner for up to 14 days despite a drop in the expression of these genes at 30 days.

### Transduction of Lentivirus Showed a Limited Influence on IPFSCs’ Stem Cell Properties

To determine whether lentivirus transduction affected IPFSCs’ proliferation and chondrogenic induction, IPFSCs were transduced with lentivirus carrying GFP followed by screening with puromycin to remove non-transduced cells (Figure 2D). PDT data showed comparable proliferation capacity in the IPFSCs with or without lentivirus transduction at passages 1, 4, and 5 following TCP expansion and at passage 5 following dECM expansion (Figure 2E). RT-qPCR data showed that, during chondrogenic induction, IPFSCs with or without lentivirus transduction had a comparable expression level of *SOX9* despite an increase of *ACAN* and a decrease of *COL2A1* in those with lentivirus transduction (Figure 2F).

### dECM Expanded IPFSCs Developed Better Premature Cartilage Tissue Constructs Than TCP Expanded Cells

Both dECM and TCP expanded IPFSCs (2.2 million each) were dynamically seeded into PLGA mesh scaffold (5 mm diameter × 2 mm thickness) in a spinner flask system. A representative tissue construct is shown in Figure 2G. Three weeks after chondrogenic induction, under microscopy, the tissue constructs seeded with dECM expanded cells appeared thicker, with cells settled on the fibers of PLGA mesh, whereas those grown with TCP expanded cells were thinner, indicating greater cell density in the dECM group than the TCP group (Figure 2H). Histology data showed that, following three-week chondrogenic induction, dECM expanded IPFSCs yielded tissue constructs with a larger size and higher intensity of sulfated GAGs as stained by Alcian blue (Ab) and type II collagen (Col 2) immunostained by monoclonal antibody (Figure 2I). These observations were further supported by RT-qPCR, as tissue constructs made by dECM expanded cells had higher expression levels of chondrogenic marker genes *SOX9*, *COL2A1*, and *ACAN* than the TCP group; interestingly, the dECM group had less expression of the hypertrophic marker gene *COL10A1* (Figure 2J).

### Early Stage Evidence of Cartilage Resurfacing Using Different Approaches

Premature tissue constructs from the dECM and TCP groups were used to fill in the defects with implantation of PLGA scaffold alone and the defect left untreated as controls. Six weeks after implantation, defects left untreated (the Empty group) exhibited the best cartilage regeneration with glistening, smooth, and whitish neotissue in most joint samples; however, in other groups, some defects remained uncovered or were partially covered with neotissue, showing a donor-dependent manner of cartilage regeneration. The best and worst examples of healed defects on both sides of the dECM group were exhibited by rabbit No. 10 and No. 7, respectively (Figure 3). Greatest healing of defects in the TCP group was found in rabbit Nos. 16 and 17, whereas healing was more limited in rabbit Nos. 15 and 18 (Figure 3). Despite lack of inflammatory signs in synovial tissue in all six-week groups, we found subchondral bone cysts in all groups and mononuclear cells in some groups (Table 2) as well as subchondral bone spurs in some rabbit joints, including rabbits Nos. 2 and 3 (left side) in the PLGA group and rabbit No. 9 in the dECM group (both left and right sides**;** Figure 3).

The above-mentioned morphological appearance of six-week cartilage resurfacing was further confirmed by histology and immunostaining (Figure 4). Most defects in the Empty group were filled with regenerated tissue having integrated at both sides and intensive staining of Alcian blue for sulfated GAGs and immunostaining for type II collagen as well as less staining of type I collagen located primarily on the surface of the neotissue, indicative of a mature articular cartilage (for example, in rabbit No. 25 on the left side). Bone spurs that were composed of regenerated tissue stained positively for sulfated GAGs and type II collagen, indicating the presence of hyaline cartilage, covered with a tissue stained positively for types I and II collagen, indicative of fibrocartilage (Figure 5). However, we also found subchondral bone cysts in some joints (No. 26, right side), which likely formed *via* an extension of regenerated cartilage; the wall of cysts expressed both types I and II collagen but not sulfated GAGs, suggestive of fibrocartilage. The other groups included the “best” healing of osteochondral defects such as rabbit No. 2 (right side) in the PLGA group, No. 10 (right side) in the dECM group, and No. 17 (right side) in the TCP group. The “worst” healing of osteochondral defects was found in rabbit No. 1 (left side) in the PLGA group, No. 7 (left side) in the dECM group, and No. 15 (left side) in the TCP group. The MODS scores (“Empty” *versus* “PLGA”, *p* = 0.009; “Empty” *versus* “dECM”, *p* = 0.000; and “Empty” versus “TCP”, *p* = 0.000) (Table 3) support the above observation, indicating that the Empty group outperformed the other implantation groups in cartilage resurfacing.

### Late Stage Evidence of Cartilage Resurfacing Using Different Approaches

The Empty group exhibited superior cartilage healing as compared to all other groups (Figure 6), which was supported by their MODS scores (“Empty” vs. “PLGA”, *p* = 0.039; “Empty” vs. “dECM”, *p* = 0.038; and “Empty” vs. “TCP”, *p* = 0.028) (Table 3). Compared to those of six-week rabbit joints, cartilage regeneration in the 15-week joints of the Empty group did not have a significant change; however, other groups at 15 weeks had greatly improved in osteochondral defect repairs, particularly for the dECM and TCP groups which had implantation of tissue constructs (Figures 6, 7).

There were no signs of inflammation or bone spurs in 15-week joints in any group. Compared to six-week cartilage resurfacing, we found more bone cysts in each group and mononuclear cells surrounding regenerated tissue in some groups (Table 2), particularly in rabbit No. 4 (left side) in the PLGA group (Figure 7). The MODS score of cartilage resurfacing with tissue constructs (dECM and TCP groups) exhibited a significant increase at 15 weeks compared to that at six weeks (*p* = 0.041 and *p* = 0.022, respectively) (Table 3).

### Tracking of Implanted Cells Labeled With GFP

Under immunofluorescence microscopy, GFP expression in both *in vitro* tissue constructs was maintained from expanded IPFSCs after lentivirus transduction and puromycin screening (Figure 8A). However, GFP expression in the regenerated cartilage tissue was undetectable in all groups at both six-week (Figure 8B) and 15-week time points (Figure 8C), indicating that implanted IPFSCs might not be directly involved in cartilage resurfacing. In order to exclude the influence of decalcification on the immunofluorescence signal, an immunohistochemical staining was conducted using a monoclonal antibody against GFP. The result confirmed immunofluorescence data (Figures 8A–C) – positive staining in *in vitro* tissue construct samples (Figure 8D) but not in *in vivo* resurfacing cartilage from the tissue construct groups (Figure 8E).

## Discussion

The goal of this study was to assess the feasibility of using a dECM-mediated-tissue engineering approach to treat osteochondral defects in young rabbits. Interestingly, we found that the Empty group (with defects left untreated) exhibited superior cartilage resurfacing at both six weeks and 15 weeks compared to the PLGA, TCP, and dECM groups. In addition, the MODS score of 15-week cartilage resurfacing in the Empty group had no significant change compared to that of six-week samples, indicating that 7.7-month-old rabbits still had a strong capacity to self-heal cartilage defects up to six weeks until 9 months of age (7.7 months + 6 weeks) by which time the rabbits had lost this ability. Consistent with a previous report, (He et al., 2009) despite the excellent chondrogenic capacity and less hypertrophy of dECM expanded IPFSCs evaluated *in vitro*, tissue constructs developed by dECM expanded cells failed to show an advantage for cartilage resurfacing over those from TCP expanded cells. However, the MODS scoring data indicated that cartilage resurfacing was significantly improved in both tissue construct groups at 15 weeks compared to those at six weeks, suggesting that a tissue-engineering approach plays a unique role in cartilage resurfacing of adult rabbits despite the fact that self-healing dominates cartilage repair in young rabbits less than 9 months old. Although the implanted cells were pre-labeled with GFP, no positive staining was detectable in the resurfaced cartilage from both six-week and 15-week osteochondral defects, suggesting that the implanted cells might not be directly involved in cartilage resurfacing.

As a conventionally used animal model, the rabbit has a strong ability for spontaneous cartilage repair, (Chu et al., 2010; Anderson et al., 2014) which implies the chondrocytes’ capacity in proliferation and deposition of functional matrix in the absence of vascular elements (Dell’Accio and Vincent, 2010). Therefore, it is important to choose rabbits with minimized self-healing capacity for a cartilage regeneration study. NZW rabbits’ skeletal maturity is reported to occur between four and six months, (Reinholz et al., 2004; Hunziker et al., 2007) but some groups believe rabbits become skeletally mature between six and nine months of age (Rudert, 2002; Isaksson et al., 2010) or between seven and eight months of age, (Masoud et al., 1986) with an age of eight months and above, (Wei et al., 1997; Wei and Messner, 1999; Pei et al., 2009) or with an age of nine months or more (Hoemann et al., 2007). The finding in this study indicates there is no further growth of cartilage when rabbits reach nine months old, the age when a young rabbit becomes an adult (Laber-Laird et al., 1996), which might be attributed to cartilage maturation, meeting the guidelines recommended by the International Cartilage Regeneration & Joint Preservation Society (ICRS), as opposed to skeletal maturity (Hurtig et al., 2011). Cartilage maturation is defined by an intact tidemark that is the calcified cartilage layer and complete subchondral bone plate with minimized vascularization (Müller-Gerbl, 1998; Madry et al., 2010). Given a 3-mm diameter cartilage lesion defined as the critical sized defect in a rabbit knee model, in this study, 4.76 mm diameter × 2 mm depth osteochondral defects that did not penetrate subchondral bone in the Empty group were filled with a neotissue with intensive expression of sulfated GAGs and type II collagen but less expression of type I collagen, indicative of a hyaline articular cartilage. These findings are in contrast to fibrocartilage with inferior mechanical properties as reported in the Empty group by Barron et al. Barron and coworkers reported that type I collagen was evident throughout the neotissue along with type II collagen, (Barron et al., 2015) likely contributed by bone marrow stomal cells released from penetrating subchondral bone through a 3-mm-depth cartilage defect model (Wei and Messner, 1999).

Some researchers think articular cartilage is immunoprivileged because of cartilage’s avascular and dense ECM; however, this view has been questioned by antigenic evidence of chondrocytes and associated ECM, (Revell and Athanasiou, 2009; Arzi et al., 2015). As shown by the cartilage resurfacing joint samples in the PLGA group, implant materials evoked a robust and constant inflammatory response evidenced by the presence of a large number of mononuclear cells surrounding subchondral bone at 15 weeks postoperatively. However, there was no sign of immune rejection observed during tissue harvesting. This finding confirmed the view that the recipient could reject a xenogeneic but not allogeneic implant (Pei et al., 2009, 2010; Arzi et al., 2015). Increasing evidence shows that the discrepancy exists in response to foreign implants between young and old recipients due to the changed local matrix microenvironment (Lynch and Pei, 2014; Brown et al., 2017). For example, Hachim et al. (2017) reported that, compared to eight-week-old mice, 18-month-old mice exhibited significant differences in macrophage polarization during the early phase of implantation and delayed resolution of the host response. Colvin et al. (2017) demonstrated that older transplant recipients exhibited reduced frequency of acute allograft rejection due to immunosenescence. The above-mentioned evidence might partially explain why implant groups were not better in cartilage resurfacing than the Empty group (left untreated), at least in the earlier time points assessed in this study, such as six weeks and 15 weeks.

Abnormal reactions during cartilage resurfacing include, but are not limited to, osteophytes, bone cysts, and synovial tissue inflammation (Hoemann et al., 2011). In this study, we did not observe synovial tissue inflammation, but both osteophytes and bone cysts existed in some groups at some time points. In animal models, subchondral bone cysts can appear following the treatment of cartilage repair, (Benazzo et al., 2008; Getgood et al., 2012) suggesting abnormal biological remodeling (Henderson et al., 2003) resulting from unusual mechanobiology (Von Rechenberg et al., 2003; Pallante-Kichura et al., 2013). Different from previous findings that bone cysts were only observed in the Empty group but not in the cell-free or cell-seeded scaffold groups (Barron et al., 2015) and that bone cysts occurred in the implantation with either collagen-GAG or PLGA scaffold, (Getgood et al., 2012) we found that subchondral bone cysts existed in all groups at both time points; however, cartilage resurfacing at 15 weeks postoperatively had more bone cysts than the earlier time point at six weeks. Since both time points designed for observation were still in the early phase of cartilage resurfacing, the wall of bone cysts was characterized as fibrocartilage, which positively stained for both types I and II collagen but was negative for sulfated GAG. This finding is in contrast to previous reports in which mature bone cysts were surrounded by bone tissue (Chen et al., 2011; Pallante-Kichura et al., 2013).

Potential mechanisms underlying the role of mesenchymal stromal/stem cells in cartilage repair include two viewpoints, *via* direct (chondrogenic differentiation) and/or indirect (secretion of paracrine factors) strategies (Meirelles Lda et al., 2009; Toh et al., 2014). Previous studies indicated that only a small fraction of labeled cells traceable in the repair tissue originated from the implanted cells (Grande et al., 1989; Dell’Accio et al., 2003; Tatebe et al., 2005; Blanke et al., 2009). In this study, we were unable to trace at either six-weeks or 15-weeks postoperatively using both immunofluorescence microscopy and immunohistochemical staining for GFP signal, indicating that trophic factors released by the implanted cells might contribute to cartilage resurfacing rather than direct differentiation. In comparison to defects at six and 15 weeks, both tissue construct groups exhibited a significant improvement in cartilage resurfacing indicting that the impact of implanted cells on reparative cells might dominate osteochondral defect repair and play a more critical role than the implanted cells themselves (Muschler et al., 2010).

Taken together, in this study, young NZW rabbits (around 7.7 months old) exhibited a strong ability for simultaneous cartilage regeneration until nine months of age. Compared to TCP expanded IPFSCs, dECM expanded cells presented a robust chondrogenic capacity under *in vitro* induction in both pellet and tissue construct cultures, but this advantage was not reflected in cartilage resurfacing of osteochondral defects in young rabbits. Interestingly, both tissue construct groups displayed improved cartilage resurfacing in a time-dependent manner, indicating that a tissue-engineering cartilage graft can facilitate osteochondral defect repair in adult rabbits, in which the untreated group did not have improvement. In the future, the dECM-based tissue-engineering approach will be further explored to treat osteochondral defects in models utilizing older animals, including adult and elderly rabbits with mature cartilage.

## Data Availability Statement

The raw data supporting the conclusions of this article will be made available by the authors, without undue reservation.

## Ethics Statement

The animal study was reviewed and approved by WVU IACUC committee.

## Author Contributions

ZL performed collection and assembly of data, data analysis and interpretation, manuscript writing, and final approval. SZ, JV, AS, and JP performed collection of data and final approval of manuscript. GG performed data analysis and interpretation and final approval of manuscript. LY performed data analysis and interpretation, final approval of manuscript, and financial support. MP performed conception and design, data analysis and interpretation, manuscript writing and final approval, and financial support. All authors contributed to the article and approved the submitted version.

## Conflict of Interest

The authors declare that the research was conducted in the absence of any commercial or financial relationships that could be construed as a potential conflict of interest.
